# Brachioradialis tendon transfer for a thumb and finger extension disorder owing to distal-type cervical spondylotic amyotrophy: a case report

**DOI:** 10.1080/23320885.2025.2610522

**Published:** 2025-12-30

**Authors:** Risa Takenaka, Takashi Oda, Tsutomu Oshigiri, Takuro Wada, Atsushi Teramoto

**Affiliations:** ^a^ Department of Orthopedic Surgery, Saiseikai Otaru Hospital, Otaru, Japan; ^b^ Department of Orthopedic Surgery, School of Medicine, Sapporo Medical University, Sapporo, Japan

**Keywords:** Brachioradialis, cervical spondylotic amyotrophy, drop finger, finger extention disorder, tendon transfer

## Abstract

To reconstruct finger extension in distal-type cervical spondylotic amyotrophy, wrist flexor tendons are commonly selected as the donor tendons. However, distal-type cervical spondylotic amyotrophy predominantly affects the C8 myotome, and therefore the wrist flexor muscles may also be impaired. The brachioradialis is mainly innervated by the C6 and is therefore less likely to be affected, it represents a favorable option as a donor tendon. However, there are few reports describing the use of the brachioradialis as a donor tendon. A 72-year-old man presented with limited active extension of his left ring and small fingers and thumb. Posterior cervical decompression was performed for cervical spondylotic amyotrophy (C8 segment involvement); however, at 12 months postoperatively, thumb and finger extension remained impaired. Instead of a wrist flexor transfer, we performed a brachioradialis tendon transfer to the extensor pollicis longus and extensor digitorum communis tendons. At 2 - year postoperative final follow-up, the extension lags of the 4^th^ and 5^th^ metacarpophalangeal joints and thumb had improved. His Disabilities of the Arm, Shoulder, and Hand score improved from 30 to 11.6 postoperatively. In finger and thumb extensor paralysis owing to distal cervical spondylotic amyotrophy, the brachioradialis muscle is typically spared and can be considered a donor for tendon transfer. This procedure, combined with extensive release of the fascial attachments, effectively improved dysfunction of finger and thumb extension without resulting in significant functional loss.

## Introduction

Cervical spondylotic amyotrophy (CSA) is a disorder caused by cervical spondylotic changes, leading to impairment of the anterior horn or ventral root and resulting in upper limb-dominant muscle weakness and atrophy [1]. Distal-type CSA often presents with finger drops, and residual paralysis contributes to hand impairment. As sensory function remains intact, tendon transfer for finger extension can improve a patient’s activities of daily living (ADL) [2]. To reconstruct finger and thumb extension in distal-type CSA, the flexor carpi radialis (FCR) or flexor carpi ulnaris (FCU) or palmaris longus (PL) tendon is commonly selected as the donor tendon, according to the surgical procedure of reconstruction for radial nerve palsy. However, distal-type CSA predominantly affects the C8 myotome, and therefore wrist flexors may also be impaired. The brachioradialis (BR) is mainly innervated by the C6 and is therefore less likely to be affected, it represents a favorable option as a donor tendon. However, there are few reports describing the use of the BR as a donor tendon. Here, we report a case of BR tendon transfer to reconstruct a finger extension disorder owing to distal-type CSA following cervical decompression surgery and its management.

## Case presentation

Ethical approval was not required for this case report, in accordance with the policy of our institution. Written informed consent was obtained from the patient for publication of this case report and any accompanying images or identifying information.

## Case: 72-year-old man (right dominant)

**Figure 1.**
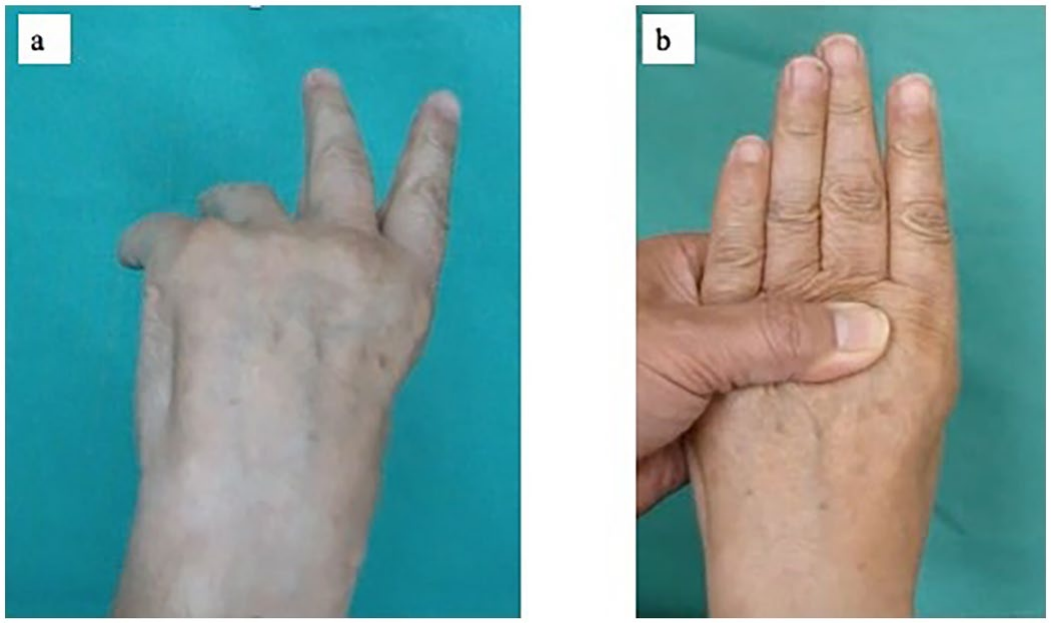
Preoperative appearance. **a**. Maximum finger active extension. The range of motion of the MP joints in active extension was −50° in the ring finger, and −70° in the little finger. **b**. Active finger extension with the MP joints held in extension. Full extension of the PIP and DIP joints can be achieved. Abbreviations: MP, metacarpophalangeal.

**Figure 2.**
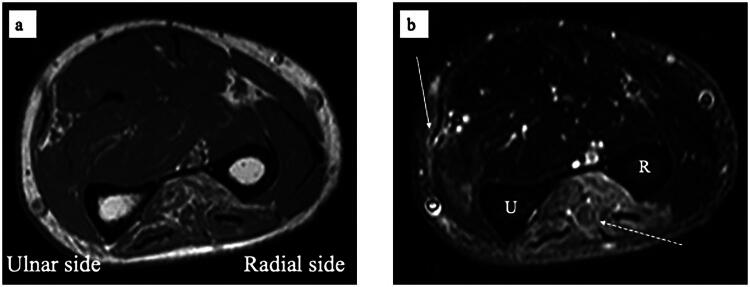
MRI of the left forearm at initial visit to the hand clinic. **a**. T1WI image, **b**. STIR image (white arrow, FCU; white dashed arrow, finger extensor group). It revealed high signal intensity in the extensor muscles and the FCU in STIR image. Abbreviations: FCU, flexor carpi ulnaris; MRI, magnetic resonance imaging; STIR, short-tau inversion recovery.

**Figure 3.**
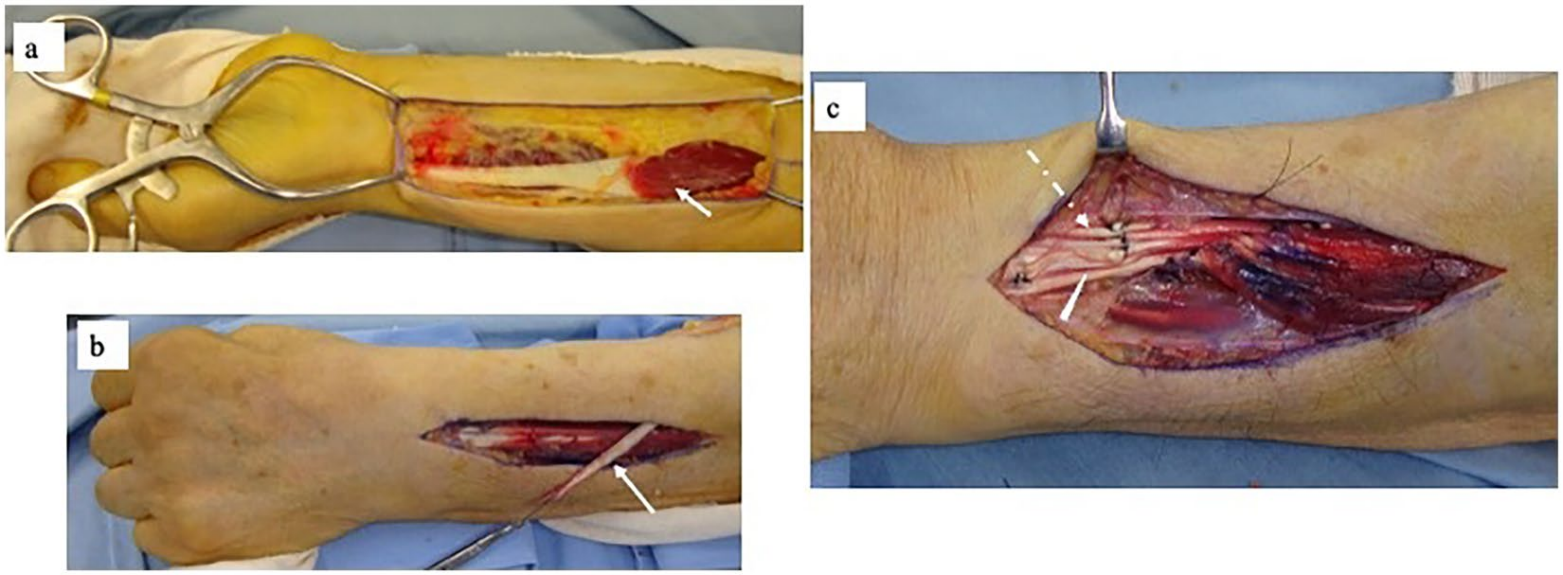
Surgical procedure of BR tendon transfer to EDC and EPL. **a**. The volar aspect of the forearm is exposed through a longitudinal incision. The distal tendon insertion of the BR (white arrow) is transected, and the muscle belly is meticulously dissected from the surrounding fascia up to the proximal one-fourth of the forearm. **b**. Through a dorsal incision, the four EDC tendons were sutured in the neutral wrist position with the MP joints in full extension (0°) using 3-0 nylon. The EPL (white dashed arrow) is sutured to the EDC (white arrowhead) with the thumb in full extension. With the elbow in flexion, the wrist in maximum extension, the MP joints at 0°, and the thumb in maximal extension, the BR was maximally tensioned and woven twice through the EPL and each of the four EDC tendons, followed by tendon-to-tendon weaving sutures. Abbreviations: BR, brachioradialis; EDC, extensor digitorum communis; EPL, extensor pollicis longus; MP, metacarpophalangeal.

A 72-year-old man was referred to our hand clinic by a spine surgeon because of extension impairment of the left thumb and fingers. The flow chart indicates the patient’s clinical course, including diagnosis and treatment details (Table 1). He had undergone posterior cervical decompression for extension deficits of the thumb and fingers due to distal-type CSA (C8 segment involvement) one year prior. Although the preoperative left-dominant spinal cord compression at the C6/7 level was resolved, extension impairment of the thumb and fingers persisted. When he visited our clinic, abductor digiti minimi muscle atrophy was evident on visual assessment. Manual muscle testing (MMT) revealed the following scores: elbow flexion, 5; elbow extension, 5; wrist extension, 5; wrist flexion, 4; metacarpophalangeal joint extension of the fingers, 2–3; thumb extension, 0–1; and radial abduction of the thumb, 0–1. He was able to actively stretch his index and middle fingers; however, extension lags were observed in the thumb and the ring and small fingers (Figure 1a). Active extension of the distal interphalangeal (DIP) and proximal interphalangeal (PIP) joints of the index finger to the little fingers was possible when the metacarpophalangeal (MP) joints were in an extended position (Figure 1b). No sensory disturbances were observed. The fingertip pinch measured 11.5 kg on the right and 7.5 kg on the left side. Grip strength was 48 kg on the right side and 30 kg on the left side, indicating reduced strength on the left side. The Japanese version of the Disabilities of the Arm, Shoulder, and Hand (DASH-JSSH) score was 30. In the Japanese version of the Michigan Hand Outcomes Questionnaire (MHQ-J), the ADL scores were 35.3 and 67.8 for the left and right hands, respectively. The total left- and right-hand scores were 43.6 and 80.4, respectively. MRI on short-tau inversion recovery images revealed a high signal intensity in the finger extensor mass and FCU (Figure 2a and b). A BR tendon transfer was performed owing to persistent finger drop resulting from C8 involvement. We followed the surgical procedure described by Zancoli [3] for reconstructing finger and thumb extensions in hands paralyzed as a result of mid-cervical spinal cord injuries (Figure 3). The BR tendon was passed subcutaneously over extensor carpi radialis longus (ECRL) and extensor carpi radialis brevis (ECRB) and was interlaced with the pre-sutured extensor pollicis longus (EPL) and extensor digitorum communis (EDC) tendons. Full MP joint extension (0°) and maximum thumb extension were observed with the elbow at 90° flexion and the wrist at 30° palmar flexion. The wrist was splinted in extension, with the thumb to the small finger held in extension for four weeks postoperatively. A sling was used during postoperative week 1. Active mobilization of the fingers and thumb in a stress-reduced position was initiated on postoperative day 1. The patient underwent rehabilitation including place-and-hold exercise, lumbrical muscle activation training, and intrinsic muscle stretching.

**Table 1.** Flowchart of the treatment process for drop fingers.

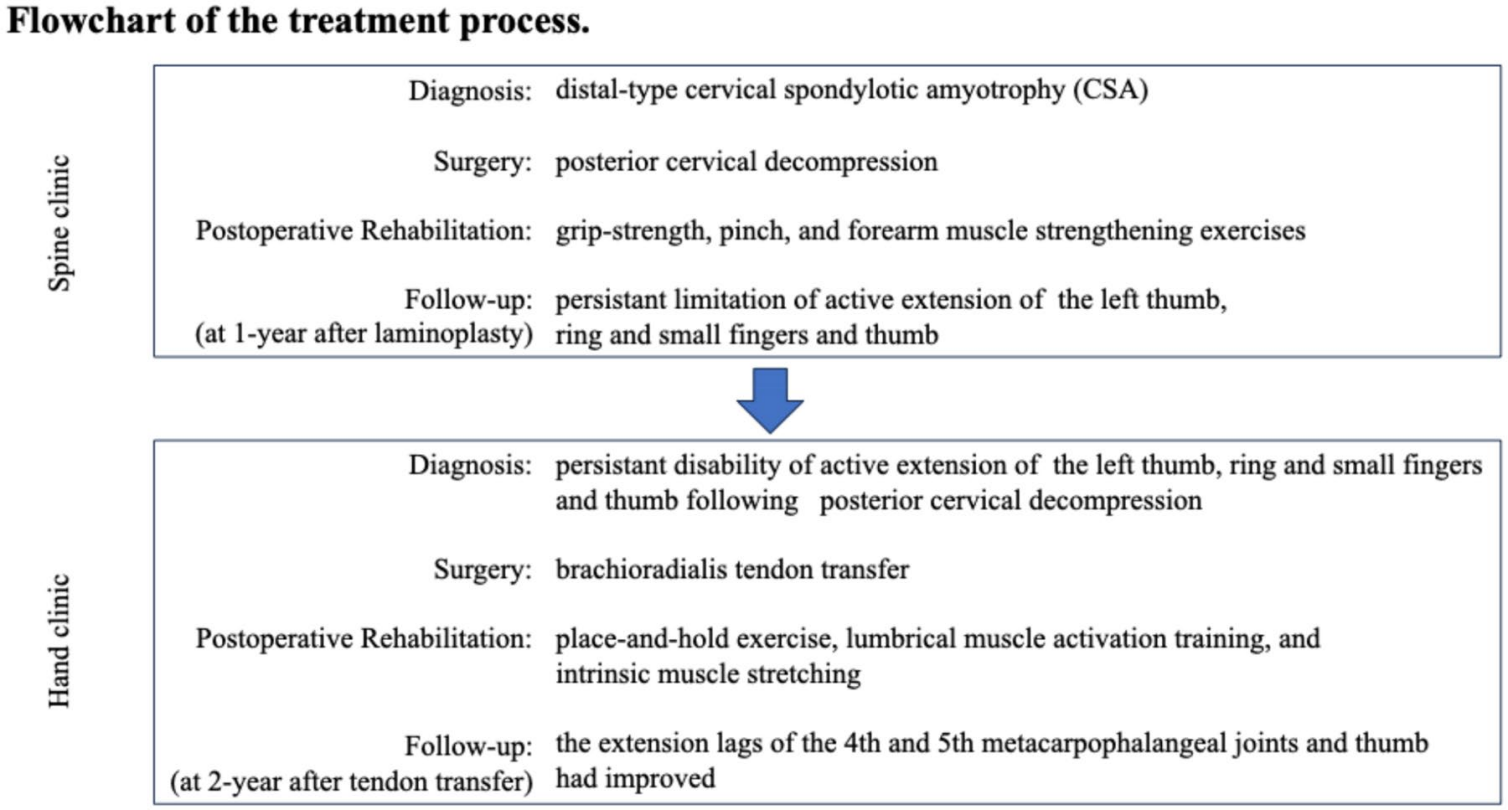

**Figure 4.**
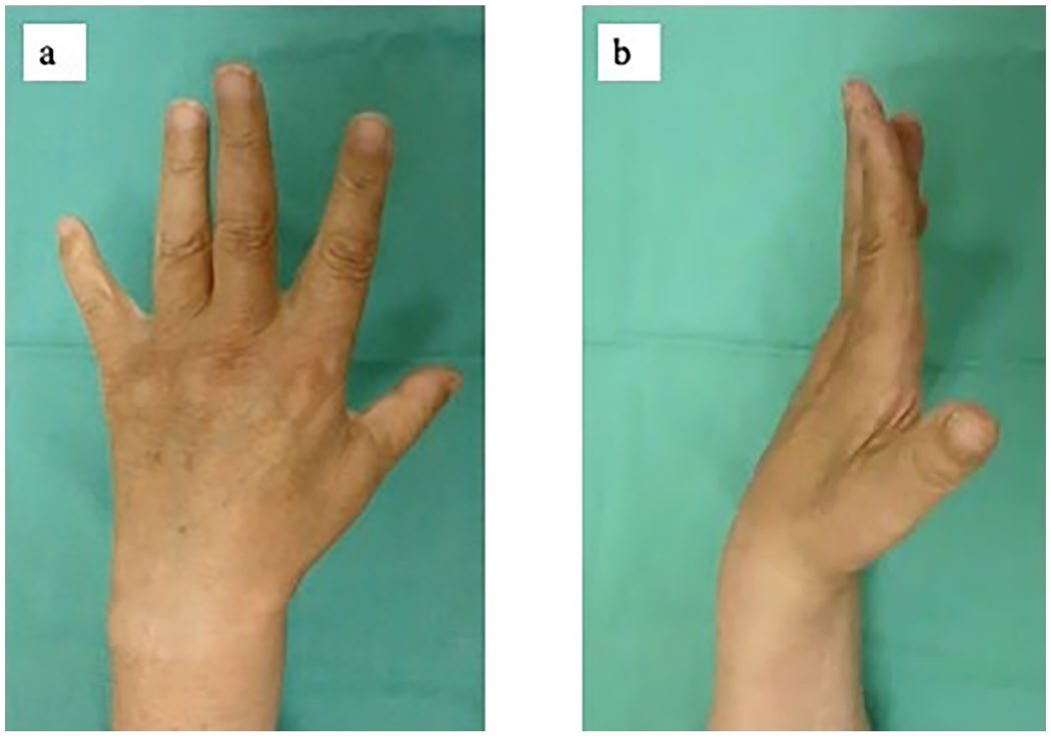
Appearance of the hand in the opening position at 2 years postoperatively. **a**. Dorsal aspect. **b**. Lateral aspect. The limitation of 4^th^ and 5^th^ MP joint and thumb extension improved, but wrist flexion was observed during finger extension.

At two years postoperatively, the extension lag of the 4^th^ and 5^th^ MP joints, as well as the thumb, had improved (Figure 4a). However, a slight wrist flexion was observed during finger extension (Figure 4b). Grip strength was 48.0 kg on the right and 30.0 kg on the left side (affected/unaffected ratio: 62.5%), with no significant change observed. The DASH-JSSH score improved from 30 to 11.6 following surgery. The MHQ-J scores also improved, with the ADL score for the left hand increasing to 66, and the total score increasing to 57.

## Discussion

In distal-type CSA, impairment is limited to the lower cervical motor neurons, allowing for reconstruction of finger extension and stabilization of the MP joints using tendon transfer with preserved muscles such as FCR, FCU and PL. However, there are two problems associated with tendon transfer using the wrist flexor muscles for distal-type CSA. As mentioned above, one is that the wrist flexor muscles can be weakened in cases of C8 palsy, potentially limiting their efficacy as donor tendons for transfer. We performed BR tendon transfer to reconstruct of thumb and finger extension. As a result, the patient regained the ability to open the hand and demonstrated improved hand function. The other problem is that it is often challenging to evaluate wrist flexor strength in isolation and, if the FCR or FCU is selected as a donor without adequate assessment of paralysis, significant impairment of wrist flexion may occur postoperatively. Our patient showed acceptable wrist flexor strength (MMT, grade 4), but MRI revealed high signal changes in the FCU. Therefore, the BR tendon was selected as the donor tendon.

Wide-awake local anesthesia without tourniquet (WALANT) surgery allows intraoperative measurement of muscle excursion, enabling assessment of the suitability of a tendon as a donor for transfer [4]. In a study that evaluated the usefulness of WALANT surgery for FCR-to-EDC tendon transfer in distal-type CSA, all nine of the study’s patients showed improved active MP joint extension postoperatively, but an average extension lag of 39° across the fingers persisted [2]. While evaluation according to muscle excursion is used in distal-type CSA, alternative options remain unclear if the wrist flexor muscles are deemed intraoperatively to be as inadequate as the donor tendons [2]. If the BR tendon is primarily selected as the donor tendon, there is no risk of donor muscle weakness and no need to perform WALANT surgery for intraoperative assessment.

A previous case report described a BR tendon-to-EDC tendon transfer to reconstruct finger extension for acquired spastic hemiplegia in an adult [5]. However, the BR tendon strongly adheres to the surrounding tissues in the forearm [6], limiting its excursion; thus, it has not been commonly used as a donor tendon. A two-stage reconstruction method was developed by Zancoli for paralyzed hands post-mid-cervical cord injury, with a theoretical basis for synergistic tendon transfers and hand biomechanics [3]. They reconstructed finger extension through simultaneously transferring the BR tendon to both the EDC and EPL; however, it has been suggested that separate tenodesis of the EDC and EPL may offer more reliable outcomes [7]. In a recent cadaveric study [8], limited excursion of the BR tendon was shown to be because of its dual attachment to the radial styloid process and forearm fascia. Extensive release of the fascial attachments significantly improved the excursion of the BR tendon, and favorable outcomes were reported in BR tendon transfer to the EPL. In our case, adequate fascial release of the BR allowed us to successfully apply the Zancoli method, achieving good finger extension supported by slight wrist flexion for a dynamic tenodesis effect. Because the BR muscle is mainly innervated by C6, it is less likely to be impaired in distal CSA. Furthermore, because the biceps and brachialis muscles that flex the elbow remain intact, the use of the BR tendon as a donor tendon is not expected to result in any significant functional deficit. However, our method has a limitation. Owing to its limited excursion and weak muscle strength, BR may be unsuitable for individuals, such as those using keyboards, who require active finger extension with wrist dorsiflexion. Our surgical technique proved to be a useful option for restoring thumb and finger extension function in cases of C8 spinal segment impairment in distal-type CSA.

## Conclusions

BR tendon transfer, combined with extensive release of the fascial attachments, effectively improved dysfunction of finger and thumb extension without resulting in significant functional loss. The BR tendon represents a reliable donor for tendon transfer to reconstruct finger extension function, serving as a suitable alternative to the FCU or palmaris longus that may be compromised in C8 palsy associated with distal-type CSA.

## Data Availability

No new data were created or analyzed in this study. Data sharing is not applicable to this article.
